# Specific requirements for translation of biological research into clinical radiation oncology

**DOI:** 10.1002/1878-0261.12671

**Published:** 2020-04-08

**Authors:** Mechthild Krause, Jan Alsner, Annett Linge, Rebecca Bütof, Steffen Löck, Rob Bristow

**Affiliations:** ^1^ German Cancer Consortium (DKTK) Partner Site Dresden Germany; ^2^ German Cancer Research Center (DKFZ) Heidelberg Germany; ^3^ OncoRay ‐ National Center for Radiation Research in Oncology Faculty of Medicine University Hospital Carl Gustav Carus Helmholtz‐Zentrum Dresden – Rossendorf TU Dresden Germany; ^4^ Department of Radiotherapy and Radiation Oncology Faculty of Medicine University Hospital Carl Gustav Carus TU Dresden Germany; ^5^ Helmholtz‐Zentrum Dresden ‐ Rossendorf Institute of Radiooncology – OncoRay Dresden Germany; ^6^ National Center for Tumor Diseases (NCT), Partner Site Dresden, Germany: German Cancer Research Center (DKFZ), Heidelberg Germany; Faculty of Medicine and University Hospital Carl Gustav Carus, Technische Universität Dresden, Dresden Germany, and; Helmholtz Association / Helmholtz-Zentrum Dresden - Rossendorf (HZDR) Dresden Germany; ^7^ Department of Experimental Clinical Oncology Aarhus University Hospital Denmark; ^8^ Translational Oncogenomics CRUK Manchester Institute and Centre Division of Cancer Sciences University of Manchester UK

**Keywords:** biomarkers, clinical trials, personalized treatment, quality, radiotherapy, translational research

## Abstract

Radiotherapy has been optimized over the last decades not only through technological advances, but also through the translation of biological knowledge into clinical treatment schedules. Optimization of fractionation schedules and/or the introduction of simultaneous combined systemic treatment have significantly improved tumour cure rates in several cancer types. With modern techniques, we are currently able to measure factors of radiation resistance or radiation sensitivity in patient tumours; the definition of new biomarkers is expected to further enable personalized treatments. In this Review article, we overview important translation paths and summarize the quality requirements for preclinical and translational studies that will help to avoid bias in trial results.

## Graphical abstract

Translation of preclinical radiobiological research into clinical radiotherapy has largely improved treatment outcomes. Indicatively, based on the results of current biological research, fractionation schedules have been optimized, and combined treatments have been developed. In this Review, we discuss translation paths that have substantially improved radiotherapy and summarize what quality requirements within preclinical and translational studies will ensure the development of unbiased clinical trials.

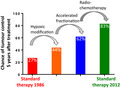

**Abbreviations**
CD44: cluster of differentiation 44 (cell surface molecule)
CXCR‐4: C‐X‐C motif chemokine receptor 4
DAHANCA: Danish Head and Neck Cancer Group
DKTK: German Cancer Consortium
EGFR: epidermal growth factor receptor
FAZA: [18F]‐fluoroazomycin arabinoside (hypoxia PET tracer)
FMISO: [18F]‐fluoromisonidazole (hypoxia PET tracer)
HNSCC: head and neck squamous cell carcinoma
HPV: human papillomavirus
PET: positron emission tomography

## 1 Introduction

Radiotherapy is, together with surgery and systemic treatments, one of the three main treatment options in oncology. Together with surgery, radiotherapy is one of the two treatment options that are able to cure solid tumours. The curative effect of radiotherapy depends on the inactivation of all cancer stem cells, that is, of those tumour cells that have an unlimited potential of cell division and thus can repopulate a tumour if surviving treatment. The potential of this subgroup of tumour cells has been described already many decades ago (Hewitt and Wilson, 1959; Steel and Stephens, 1983) in radiobiological research, although at this time no markers existed to identify stem‐cell‐rich subpopulations in tumours. Parameters that impact sensitivity of tumour cells to fractionated radiotherapy^1^Application of radiotherapy not at once, but in several sessions over usually several weeks., including overall treatment time, dose per treatment fraction, and the interval between fractions, are considered in the design of fractionation treatment schedules,. With the advent of molecular techniques and methods to identify molecular markers, molecular or cell‐based treatment options evolved, and radiobiological and translational radiooncological research developed into new areas allowing for better characterization of tumours and the development of personalized treatments, not only through the modification of radiation parameters, but also through combined treatment approaches.

## 2 Biological research with a potential to improve clinical radiotherapy

The following parameters are examples of a currently long list of putative biomarkers and potential targets for combined radiotherapy treatment schedules. We selected parameters that are relatively far developed.

### 2.1 Human papillomavirus

The infection with oncogenic viruses has been identified to be involved in the development of various cancers. For head and neck squamous cell carcinomas (HNSCC), it has been shown that the infection with the human papillomavirus (HPV) is another risk factor for the development of the disease in addition to the well‐accepted risk factors smoking and alcohol consumption. Moreover, it has been shown in a number of preclinical and clinical studies that tumours that are driven by HPV are more radiosensitive than HPV‐negative tumours, which is associated with impaired DNA repair (reviewed in Lassen (2010)). This suggests that patients with HPV‐positive HNSCC are being overtreated with the standard therapy and reduction in dose could lead to reduced toxicity. Therefore, a number of clinical trials are currently investigating if the reduction of radiation dose leads to a similar high local tumour control but less long‐term side effects due to dose de‐escalation (www.clinicaltrials.gov; e.g. NCT01088802, NCT01530997, NCT03396718).

### 2.2 Hypoxia

Numerous preclinical and clinical studies have confirmed that hypoxic tumours are associated with reduced radiation sensitivity and, consequently, poor response rates to radiotherapy (e.g. Nordsmark *et al.*, 2005; Yaromina *et al.*, 2010; Zips *et al.*, 2011). Hypoxia also predicts for distant spread of metastases and is therefore a negative prognostic factor (Bristow and Hill, 2008). *In vivo* analyses have shown that pretherapeutic tumour hypoxia significantly impacts local tumour control after radiotherapy (Yaromina *et al.*, 2010), which underlines its possible value as prognostic biomarker.

The strong progress in development of biological and functional imaging enables investigation of tumour hypoxia in a noninvasive way. For example, [18F]‐fluoromisonidazole (FMISO) or [18F]‐fluoroazomycin arabinoside (FAZA) as specific tracers for hypoxia imaging in positron emission tomography (PET) can be used in combination with computed tomography in patients with head and neck cancer or other tumour entities. In a prospective study on patients with locally advanced squamous cell carcinoma of the head and neck, a strong association of FMISO‐measured hypoxia after the first two weeks of treatment with local progression‐free survival after radiochemotherapy was demonstrated (Lock *et al.*, 2017; Zips *et al.*, 2012). Similar correlations between hypoxia and outcome of radiotherapy were obtained in further patient cohorts with FMISO‐PET (Wiedenmann *et al.*, 2015) and FAZA‐PET (Mortensen *et al.*, 2012). These clinical trials are the basis for first attempts in personalized treatment prescription (e.g. dose‐escalation trials in high‐risk patients), but also for personalized combined drug treatment with hypoxic modifiers (Overgaard, 2011).

In addition to the improvement of biological imaging, molecular investigations of tumour specimens for the assessment of hypoxia‐associated or hypoxia‐induced gene expression changes are being extensively carried out. Initial data suggest a predictive role of such hypoxia‐induced gene signatures for the outcome of patients with HNSCC and other tumours (e.g. Eustace *et al.*, 2013; Toustrup *et al.*, 2011). A prospective trial is ongoing to validate these promising results (clinicaltrials.gov NCT02661152).

### 2.3 Cancer stem cells

As mentioned above, the curative effect of radiotherapy depends on the inactivation of all cancer stem cells, that is those tumour cells that are able to re‐grow and cause a tumour recurrence (Baumann *et al.*, 2008; Clarke *et al.*, 2006). Due to the relevance of this subpopulation for local tumour control, it is aimed to integrate putative cancer stem cell markers in prognostic and predictive tests for detection and monitoring of disease.

In a retrospective analysis of cluster of differentiation 44 (cell surface molecule) (CD44) mRNA and CD44 protein expression, as putative stem cell markers on tumour material from patients with early laryngeal carcinoma, the intertumoural heterogeneity of the stem cell density as an important factor for local tumour control after radiotherapy could be shown for the first time in a clinical dataset (de Jong *et al.*, 2010). In a multicentre biomarker study of the German Cancer Consortium Radiation Oncology Group (DKTK‐ROG), CD44 protein expression has been shown to be a prognostic biomarker in patients with locally advanced HNSCC after primary radiochemotherapy (Linge *et al.*, 2016b) as well as after oncological resection followed by postoperative radiochemotherapy (Linge *et al.*, 2016a), with CD44 high expressing tumours being at high risk for tumour recurrence.

### 2.4 Growth factor receptors

The therapeutic outcome of irradiation can be negatively influenced by the induction of proliferative signalling pathways that lead to an increased tumour repopulation with cancer cells. The family of epidermal growth factor receptors (EGFR) is crucially involved in this process. Overexpression of the EGFR is frequently identified in a variety of tumour entities, and several strategies have been developed to target EGFR in various tumours, including EGFR‐specific monoclonal antibodies (such as cetuximab) and tyrosine kinase inhibitors (such as erlotinib, which block the intracellular component of the receptor). Some clinical data have shown promising results for radiotherapy combined with EGFR‐targeting treatments: combined treatment of patients with locally advanced HNSCC with cetuximab plus radiotherapy significantly improved overall survival at 5 years, as compared to radiotherapy alone (Bonner *et al.*, 2006). However, the conferred advantage of the combination of radiotherapy with cetuximab is not superior to current standard treatment (radiochemotherapy) and may even increase some toxicities (Ang *et al.*, 2014). This highlights the need for using specific biomarkers to select patients that will mostly benefit from such a combination therapy. Promising preclinical data suggest specific gene expressions as potential biomarkers for personalized combination of cetuximab with radiotherapy (Koi *et al.*, 2017).

### 2.5 Immune parameters

More recently, the impact of the immune system on radiotherapy responses and possible combinations of immunotherapy with radiotherapy has moved into the focus of translational studies. For details, see Mondini *et al.*, 2020. Especially so‐called immune‐checkpoint inhibitors showed first promising clinical results also in patients with metastatic disease. In a curative setting in patients with stage III non‐small‐cell lung carcinoma, who did not have disease progression after two or more cycles of platinum‐based chemoradiotherapy, durvalumab has been investigated as consolidation therapy compared with placebo (Antonia *et al.*, 2017). Durvalumab is a selective, monoclonal antibody that blocks programmed death‐ligand 1 binding to programmed death 1 and cluster of differentiation 80 (cell surface molecule), resulting in T cells recognizing and killing tumour cells. In this randomized phase III study, 713 patients have been included. Progression‐free survival was significantly longer with durvalumab compared to placebo, and safety was similar between the groups (Antonia *et al.*, 2017). The evaluation of long‐term overall survival data within the same trial confirmed the advantage of durvalumab (Antonia *et al.*, 2018). Further ongoing trials investigate also simultaneous treatment approaches in radiotherapy settings since there is currently a lack of evidence.

## 3 Putting biological research into context: a translational example

**Fig. 1:**
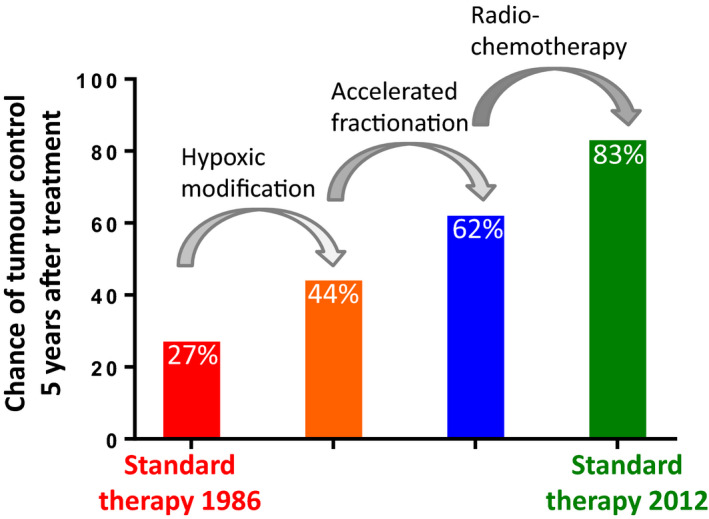
Integration of radiobiological knowledge to counteract radiotherapy resistance, showing how chance of tumour control 5 years after treatment has improved through sequential randomized clinical trials by the DAHANCA on patients with HNSCC (stage 3–4 laryngeal and pharyngeal cancer) (Baumann *et al.*, 2016).

An example of a population‐based approach to improve the outcomes of radiotherapy by integrating radiobiological knowledge is the sequence of randomized clinical trials over the past decades by the Danish Head and Neck Cancer Group (DAHANCA; Fig. 1; reviewed in (Baumann *et al.*, 2016)). In the 1980s, tumour control was achieved in ~ 30% of a specific group of HNSCC patients treated with radiotherapy. The first step was to reduce the negative impact of hypoxia with the successful introduction of the hypoxic cell radiosensitizer nimorazole. Next, the overall treatment time (time from first to last fractionation) was reduced to overcome regrowth of cancer stem cells during treatment. This was achieved by accelerated fractionation, that is by giving more fractions per week and reducing total number of weeks. Finally, chemotherapy was added to the hypoxic modification and the use of accelerated fractionation, and tumour control is now more than 80% in this group of HNSCC patients (Fig. 1). Other factors, like technological improvements in identifying the tumour targets, have certainly contributed (for details, see Fiorino *et al*., 2020), but the counteraction of biological factors of radiotherapy resistance has played a major role in the impressive improvements over time.

Setting up on the optimization of fractionation and combined treatments, individualization of radiotherapy is another important strategy to improve treatment outcome. An example is the evaluation of biomarkers for personalized radio(chemo)therapy in head and neck cancer by the ROG of the DKTK. Within this multi‐institutional retrospective–prospective study, a retrospective cohort of patients with primary or postoperative radiochemotherapy was set up. The multi‐institutional approach allowed very strong inclusion criteria, leading to a very high homogeneity of the patient parameters in the cohort. After centralized processing of tumour material, hypothesis‐based evaluation of multiple candidate biomarkers was performed by the participating centres. HPV positivity was confirmed as a strong biomarker for both primary and postoperative radiochemotherapy (Linge *et al.*, 2016b; Lohaus *et al.*, 2014). All biomarker data were fed into a joint biostatistical analysis that supported a prognostic value of a combination of tumour size, HPV status, hypoxia‐related gene expression and putative cancer stem cell markers for treatment with primary radiochemotherapy. In addition, genetic variants, that is DNA‐repair‐related single nucleotide polymorphisms and copy number variations, and specific tumour cell‐specific receptor expressions [C‐X‐C motif chemokine receptor 4 (CXCR‐4)] may increase the predictive value of the mentioned biomarker set (in preparation). These results will be validated in a prospective cohort that has already finished recruitment and can be analysed for treatment results in 2020. In parallel, HPV status as a marker for radiosensitivity of tumour cells is currently evaluated in an interventional trial, applying reduced radiation doses in postoperative radio(chemo)therapy schedules in HPV‐positive oropharyngeal cancer patients (clinicaltrials.gov NCT03396718). If successful, this approach can provide a basis for reducing the currently high severe late toxicity rate in this group of patients while keeping cure rates constant.

## 4 Coupling biological research with radiotherapy: how to avoid negative clinical trials

The fact that currently most phase II clinical trials have had negative results may rely on various reasons. One major issue is the quality of data generated preclinically before initiation of a clinical trial. To set up a valid hypothesis for an early clinical trial, preclinical‐translational experiments need to be performed as closely as possible to the clinical situation, concerning endpoints, radiation treatment schedule, potential standard for combined treatments and physical quality assurance for radiation dose and dose distribution (Coleman *et al.*, 2016). A frequently used preclinical *in vivo* endpoint allowing relatively high throughput for screening or mechanistic evaluations is tumour growth delay, that is the time that tumours need after treatment to reach a multiple of their starting volume, compared to standard treatment. However, it is known that especially in combined treatment schedules, this endpoint does not necessarily predict the curative potential of a treatment (Krause *et al.*, 2006). Thus, before start of a clinical trial aiming at an improved curative effect of radiotherapy, a local tumour control assay should be performed measuring the dose that is needed to reach permanent local control in 50% of the tumours (TCD_50_). This assay is time‐, labour‐ and cost‐intensive, but excludes many combined treatments by proving nonsuperiority over standard treatment despite promising tumour growth delay data (Krause *et al.*, 2006). Beyond the experimental methods, another issue is the quality assurance of preclinical trials. To reduce the risk of bias, specific quality measures need to be considered, including sample size calculations, inclusion and exclusion criteria, randomization, blinded assessment of outcome, and reporting of negative results (Macleod *et al.*, 2015). Homogeneity of preclinical treatments has to be assured by documentation of all treatments and doses, by exclusion rules in case of mistakes, and by physical assurance of homogeneous irradiation doses by regular dosimetry measurements at the experimental irradiation machine. A stringent guideline for publication of *in vivo* results (ARRIVE) is available with recommendations for content that should be provided to insure unbiased information of the reader (Kilkenny *et al.*, 2010).

Also, translational clinical trials on biomarkers have to follow stringent quality criteria. In a first phase of a translational study, biological factors related to treatment outcome may be identified on retrospectively or prospectively collected data. Here, false‐positive results have to be avoided, for example by estimating bias due to patient selection, heterogeneous procedures in collection, storage and analysis of biologic material, differences in treatment and follow‐up between the participating centres. Also, multiple‐testing corrections and validation approaches should be applied on datasets with a larger number of analysed parameters. In particular, complex modelling strategies for biomarker identification and outcome prediction may lead to overly optimistic results in model development, since they adapt too strongly to the given data and are thus not generalizable to new datasets. Hence, the generalizability of the results has to be tested, for example using cross validation or, even better, in validation on external multicentre datasets (Collins *et al.*, 2015; Schulz *et al.*, 2010). In addition, a second, prospective validation may be helpful to further reduce the risk of biased results. After successful validation, an interventional clinical trial may be conducted to test the efficacy of one or more defined treatment modifications for patient groups that are selected based on the most promising biomarker signatures defined in the previous analyses. Stratified block randomization, taking the most important confounders into account, should be applied, and double blinding should be preferred in case the effect of novel drugs is investigated. To avoid negative trials, a suitable primary endpoint has to be defined and the final statistical test has to be correctly chosen, accounting for competing risks, censored data and patient dropout. In addition, a realistic estimate of the expected effect and variability in the primary endpoint is decisive. Monitoring should be performed in accordance with good clinical practice including site initiation, interim monitoring and closeout. Standard operating procedures have to be clearly defined, and procedures for data acquisition and storage need to be homogenized between participating centres in order to avoid site‐specific bias and missing data. Advanced biomarker‐specific trial designs are available that may enhance the success probability of the trial and combine the steps described above. This should provide increased personalization and success when translated into ‘real‐world outcome’ studies in populations with varying comorbidity (Antoniou *et al.*, 2017, 2018; Lin and He, 2015). Specifically for clinical trials on combined radiotherapy and molecular targeted drugs, one of the best‐known examples for a lack of wide clinical implementation (even despite a positive phase III trial) was on combined radiotherapy and the anti‐EGFR antibody cetuximab (Bonner *et al.*, 2006). In this specific case, there has been a preclinical *in vivo* study on TCD_50_ for single dose irradiation before, showing a major improvement of local tumour control after combined treatment versus irradiation alone (Milas *et al.*, 2000). The clinical trial also showed superiority of the combined treatment over radiotherapy alone; however, superiority over standard combined radiochemotherapy could not be shown in later analyses (Caudell *et al.*, 2008). The fact that standard treatment changed from radiotherapy to radiochemotherapy between preparation of the protocol and final data analysis was likely not the only reason for this. More importantly, there is a heterogeneity of efficacy of the treatment between tumours of the same origin and histology. Potential predictive biomarkers as a result of extensive *in vivo* experiments with different tumour models have been described later on (without validation so far) (Koi *et al.*, 2017). Biomarker stratification or biomarker‐based treatment decisions appear as an important part of clinical trials on novel combined treatment concepts with molecularly targeted agents. Ideally, such biomarkers should be defined preclinically, using the combined treatment that is to be tested in the clinical trial compared to the standard clinical treatment, or, if available, within early clinical studies or retrospective clinical data. If a treatment decision bases on the biomarker, the marker needs to be validated (see above).

For prediction model studies, the TRIPOD statement, which gives a checklist of 22 items, deemed essential for transparent reporting of such a study, can be seen as state‐of‐the‐art reporting guideline (Collins *et al.*, 2015). For randomized clinical trials, the CONSORT statement gives similar guidance (Schulz *et al.*, 2010). Box 1 gives an overview on issues to be considered for quality assurance in preclinical and clinical trials.

Box 1 **Preclinical and translational quality assurance in radiooncological studies**

Preclinical:

- Choose treatment schedule and endpoint close to clinical situation.
- Perform sample size and power calculations.
- Define inclusion and exclusion criteria.
- Use randomized designs and blinded assessment of outcome.
- Document all treatments, define exclusion criteria in case of experimental mistakes, and assure homogeneous irradiation dose (dosimetry).
- Publish negative data.
- Consider ARRIVE guidelines for publication (Kilkenny *et al.*, 2010).

Translational:

- Consider TRIPOD statement for biomarker data (Collins *et al.*, 2015).
- Consider CONSORT statement for randomized data (Schulz *et al.*, 2010).
- Assure homogeneous patient treatment and procedures for biomaterial collection and processing.
- Conduct interventional clinical trial based on a hypothesis defined in validated trials.
- Use stratified block randomization and double‐blinded designs, whenever possible.
- Define a meaningful primary endpoint and choose an adequate statistical test.
- Use homogeneous procedures between centers for data acquisition, biomaterial storage etc.
- Perform regular monitoring during the trial and before data analysis

## 5 Future strategies and conclusion

Translation of preclinical radiobiological knowledge into clinical radiotherapy treatment schedules has largely improved outcome of radiotherapy or combined treatments over the last decades. This includes the optimization of fractionation schedules based on the results of current biological research, and, partly, also of combined treatment schedules. Personalization of treatments will lead to another major advantage. This will at a first step involve the definition of patient groups based on biological risk factors, for which a very good or a very poor predicted outcome after standard treatment is expected, with the treatment being adapted to identified and validated biomarkers. To further develop such approaches into true individual treatment decisions, learning data will help to support treatment decisions in clinical trials. Such databases use parameters of the individual patient and features of the tumour to suggest an individual treatment. Treatment outcome data are later re‐fed into the database to further optimize the prediction model. Along with individualization of radiation doses or, potentially, fractionation schedules, a major research field is still the combination of radiotherapy with systemic treatments, including molecular targeted drugs or immunotherapy, for which in many cases resistance mechanisms as well as biomarkers are currently not well known.

## Conflict of interest

In the past 5 years, Mechthild Krause received funding for her research projects by IBA (2016), Merck KGaA (2014–2018 for preclinical study; 2018–2020 for clinical study) and Medipan GmbH (2014–2018). She is involved in an ongoing publicly funded (German Federal Ministry of Education and Research) project with the companies Medipan, Attomol GmbH, GA Generic Assays GmbH, Gesellschaft für medizinische und wissenschaftliche genetische Analysen, Lipotype GmbH and PolyAn GmbH (2019–2021). For the present manuscript, Dr. Krause confirms that none of the above‐mentioned funding sources were involved. Annett Linge is involved in an ongoing publicly funded (German Federal Ministry of Education and Research) project with the companies Medipan, Attomol GmbH, GA Generic Assays GmbH, Gesellschaft für medizinische und wissenschaftliche genetische Analysen, Lipotype GmbH and PolyAn GmbH (2019–2021). For the present manuscript, Dr. Linge confirms that this above‐mentioned funding source was not involved. Jan Alsner is listed as co‐inventor on a patent for a method of determining clinically relevant hypoxia in cancer (WO/2012/146259) owned by Aarhus University, Aarhus, Denmark. The other authors confirm no conflict of interest.
